# 8-Bromo-3-(cyclo­propanylcarbon­yl)-5-methyl­indolizine-1-carbonitrile

**DOI:** 10.1107/S1600536812023161

**Published:** 2012-05-26

**Authors:** Dahe Fan, Fan Tang, Wei Wang

**Affiliations:** a Key Laboratory for Advanced Technology in Environmental Protection of Jiangsu Province, School of Chemical and Biological Engineering, Yancheng Institute of Technology, No. 9, Yingbin Avenue, Yancheng 224051, People’s Republic of China

## Abstract

The asymmetric unit of the title compound, C_14_H_11_BrN_2_O, contains three independent mol­ecules with very similar geometries. The dihedral angles between the side chain of the cyclo­propyl plane and the five-membered ring to which it is attached are 55.0 (2), 58.1 (2) and 60.2 (3)° for the three mol­ecules. Each mol­ecule forms an intra­molecular C—H⋯O hydrogen bond.

## Related literature

For background to indolizines, see: Sippl (2002 ▶); Sriram *et al.* (2005 ▶); Shen *et al.* (2007 ▶); Wu *et al.* (2011 ▶). For related structures, see: Shen *et al.* (2010 ▶). For bond-length data, see: Allen *et al.* (1987 ▶).
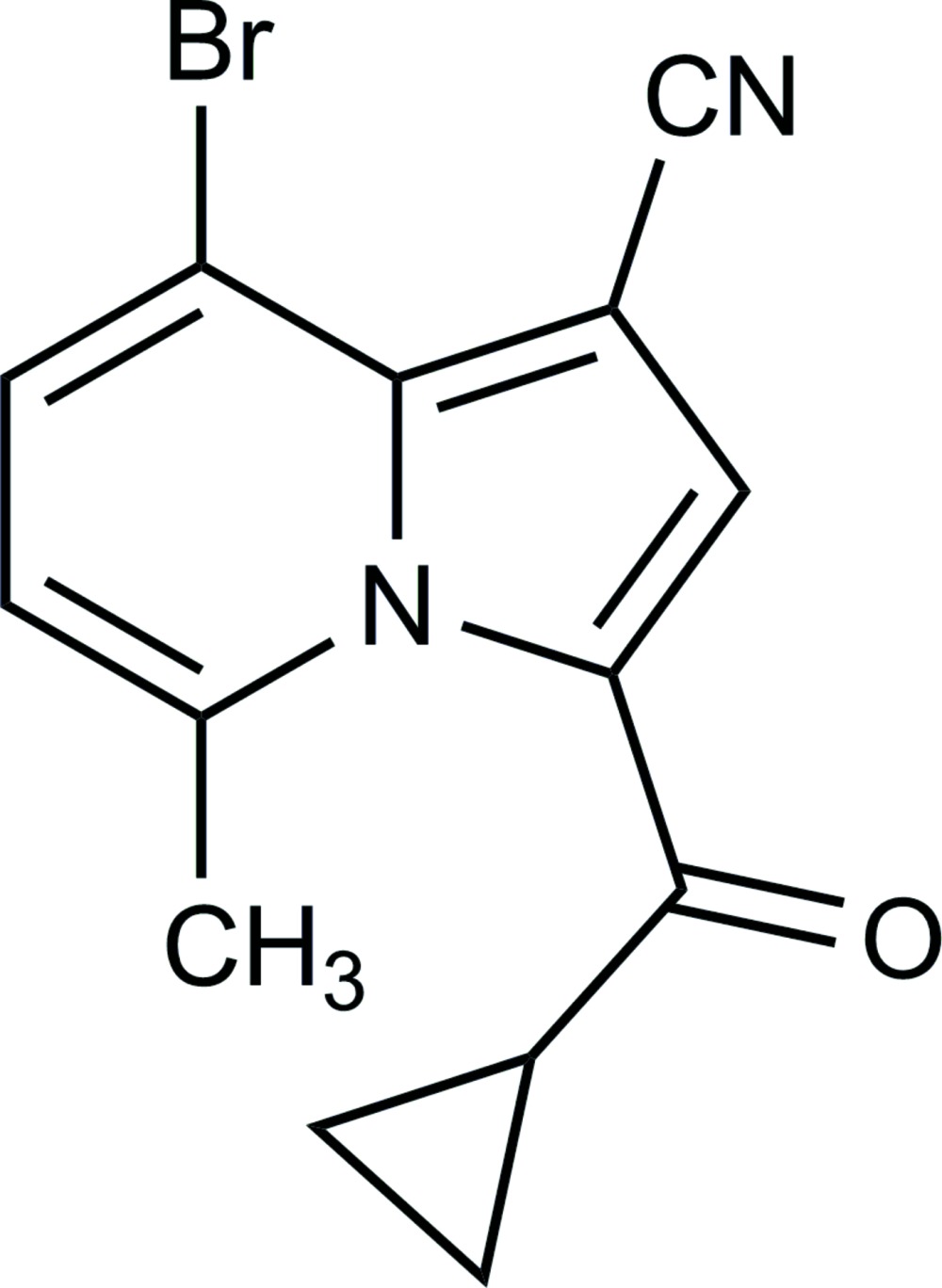


## Experimental

### 

#### Crystal data

C_14_H_11_BrN_2_O
*M* *_r_* = 303.16
Triclinic, 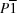

*a* = 8.3443 (7) Å
*b* = 14.5827 (13) Å
*c* = 16.9132 (14) Å
α = 70.418 (4)°
β = 88.849 (4)°
γ = 76.833 (4)°
*V* = 1884.5 (3) Å^3^
*Z* = 6
Mo *K*α radiation
μ = 3.26 mm^−1^
*T* = 296 K
0.48 × 0.33 × 0.11 mm

#### Data collection

Bruker APEXII diffractometer
Absorption correction: multi-scan (*SADABS*; Sheldrick, 1997 ▶) *T*_min_ = 0.283, *T*_max_ = 0.701
22759 measured reflections
6586 independent reflections
5163 reflections with *I* > 2σ(*I*)
*R*_int_ = 0.021

#### Refinement

*R*[*F* ^2^ > 2σ(*F* ^2^)] = 0.032
*wR*(*F* ^2^) = 0.086
*S* = 1.05
6586 reflections
487 parameters
H-atom parameters constrained
Δρ_max_ = 0.70 e Å^−3^
Δρ_min_ = −0.57 e Å^−3^

### 

Data collection: *APEX2* (Bruker, 2006 ▶); cell refinement: *SAINT* (Bruker, 2006 ▶); data reduction: *SAINT*; program(s) used to solve structure: *SHELXTL* (Sheldrick, 2008 ▶); program(s) used to refine structure: *SHELXL97* (Sheldrick, 2008 ▶); molecular graphics: *SHELXTL*; software used to prepare material for publication: *SHELXTL*.

## Supplementary Material

Crystal structure: contains datablock(s) I, global. DOI: 10.1107/S1600536812023161/fj2561sup1.cif

Structure factors: contains datablock(s) I. DOI: 10.1107/S1600536812023161/fj2561Isup2.hkl

Supplementary material file. DOI: 10.1107/S1600536812023161/fj2561Isup3.cml

Additional supplementary materials:  crystallographic information; 3D view; checkCIF report

## Figures and Tables

**Table 1:** Hydrogen-bond geometry (Å, °)

*D*—H⋯*A*	*D*—H	H⋯*A*	*D*⋯*A*	*D*—H⋯*A*
C14—H14*B*⋯O1	0.96	2.09	2.857 (4)	136
C28—H28*C*⋯O2	0.96	2.04	2.823 (4)	138
C42—H42*B*⋯O3	0.96	2.04	2.814 (4)	136

**Fig. 1.**
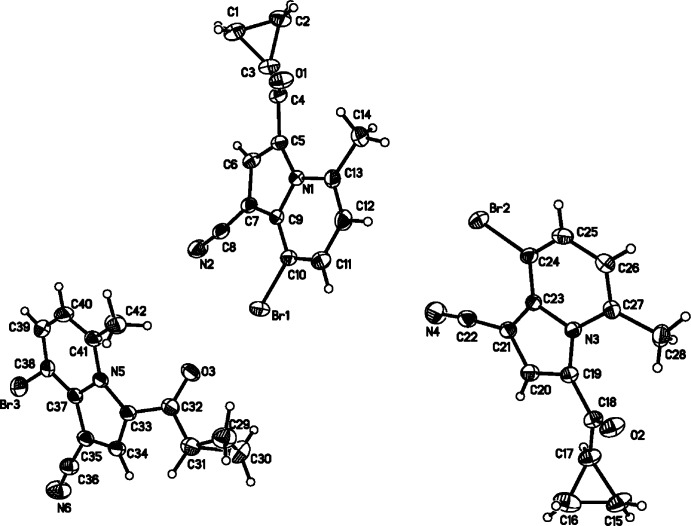
The molecular structure of the title compound, with 30% probability displacement ellipsoids. Dashed lines indicate hydrogen bonds.

## supplementary crystallographic information

### Comment

Indolizines have been drawing greatly research interest owing to their special electronic structures high fluorescence efficiency (Shen *et al.*, 2007), wide range of biological activity (Sippl, 2002; Wu *et al.*, 2011) and the fact that their partially hydrogenated frameworks have been found in several natural products (Sriram *et al.*, 2005). Meanwhile, the cyclopropane is a noteworthy structural motif because of its own biological activity and its effect to modify the bioactivity of the parent compounds(Shen *et al.*, 2010). We report here the synthesis and crystal structure of the title indolizine compound which is containing cyclopropylcarbonyl group.

The molecular structure of the title compound is shown in Fig. 1. In the title compound, The distance of C—Br bond for these three molecules is 1.883 (3), 1.879 (3), 1.887 (3) Å respectively, similar to the value of 1.882 (3) Å found in the previously reported structure [Wu *et al.* (2011)]. The values of the geometric parameters for the others in the structure are normal (Allen *et al.*, 1987). the indolizine plane is slightly twisted, with maximum deviations of 5.979 (7), 7.111 (8), and 7.0657 Å from the mean plane. The dihedral angle between the side chain of the cyclpropyl plane and the five-membered ring to which it is attached is 54.98 (20)°, 58.10 (21)° and 60.18 (30)° for the three molecules in the asymmetric unit. Each molecule forms an intramolecular C–H.. O hydrogen bond.

### Experimental

A mixture of the 5-bromo-1-(2-cyclopropyl-2-oxoethyl)-2-methylpyridinium bromide salt (10 mmol), acrylonitrile (40 mmol), triethylamine (2 ml) and TPCD (4 g) in DMF (40 ml) was heated at 90° C for 5 h. After cooling, the reaction mixture was poured into an aqueous hydrochloric acid solution (5%, 100 ml), the precipitated crude product was collected by filtration and further purified by silica gel column chromatography with petroleum ether (bp 60–90 °*C*)-ethyl acetate as eluents. Yellow crystal. m.p.133–135 °C, Yield 75%. Single crystals suitable for X-ray diffraction were prepared by slow evaporation of a solution of the title compound in petroleum ether -ethyl acetate(5 mL:1 mL), at room temperature.

### Refinement

The H atoms were fixed geometrically and were treated as riding on their parent C atoms, with C—H distances in the range of 0.93–0.97 Å, and with *U*_iso_(H) = 1.2*U*_eq_(parent atom), or *U*_iso_(H) = 1.5*U*_eq_(C_methyl_).

### Crystal data

**Table:**

C_14_H_11_BrN_2_O	*Z* = 6
*M**_r_* = 303.16	*F*(000) = 912
Triclinic, *P*1	*D*_x_ = 1.603 Mg m^−^^3^
Hall symbol: -P 1	Mo *K*α radiation, λ = 0.71073 Å
*a* = 8.3443 (7) Å	Cell parameters from 9236 reflections
*b* = 14.5827 (13) Å	θ = 1.5–25.0°
*c* = 16.9132 (14) Å	µ = 3.26 mm^−^^1^
α = 70.418 (4)°	*T* = 296 K
β = 88.849 (4)°	Block, brown
γ = 76.833 (4)°	0.48 × 0.33 × 0.11 mm
*V* = 1884.5 (3) Å^3^	

### Data collection

**Table:**

Bruker APEXII diffractometer	6586 independent reflections
Radiation source: fine-focus sealed tube	5163 reflections with *I* > 2σ(*I*)
Graphite monochromator	*R*_int_ = 0.021
ω scans	θ_max_ = 25.0°, θ_min_ = 1.5°
Absorption correction: multi-scan (*SADABS*; Sheldrick, 1997)	*h* = −9→9
*T*_min_ = 0.283, *T*_max_ = 0.701	*k* = −17→15
22759 measured reflections	*l* = −20→20

### Refinement

**Table:**

Refinement on *F*^2^	Primary atom site location: structure-invariant direct methods
Least-squares matrix: full	Secondary atom site location: difference Fourier map
*R*[*F*^2^ > 2σ(*F*^2^)] = 0.032	Hydrogen site location: inferred from neighbouring sites
*wR*(*F*^2^) = 0.086	H-atom parameters constrained
*S* = 1.05	*w* = 1/[σ^2^(*F*_o_^2^) + (0.0481*P*)^2^ + 0.4575*P*] where *P* = (*F*_o_^2^ + 2*F*_c_^2^)/3
6586 reflections	(Δ/σ)_max_ = 0.001
487 parameters	Δρ_max_ = 0.70 e Å^−^^3^
0 restraints	Δρ_min_ = −0.57 e Å^−^^3^

### Special details

**Table:**

Geometry. All e.s.d.'s (except the e.s.d. in the dihedral angle between two l.s. planes) are estimated using the full covariance matrix. The cell e.s.d.'s are taken into account individually in the estimation of e.s.d.'s in distances, angles and torsion angles; correlations between e.s.d.'s in cell parameters are only used when they are defined by crystal symmetry. An approximate (isotropic) treatment of cell e.s.d.'s is used for estimating e.s.d.'s involving l.s. planes.
Refinement. Refinement of *F*^2^ against ALL reflections. The weighted *R*-factor *wR* and goodness of fit *S* are based on *F*^2^, conventional *R*-factors *R* are based on *F*, with *F* set to zero for negative *F*^2^. The threshold expression of *F*^2^ > σ(*F*^2^) is used only for calculating *R*-factors(gt) *etc*. and is not relevant to the choice of reflections for refinement. *R*-factors based on *F*^2^ are statistically about twice as large as those based on *F*, and *R*- factors based on ALL data will be even larger.

### Fractional atomic coordinates and isotropic or equivalent isotropic displacement parameters (Å^2^)

**Table:**

	*x*	*y*	*z*	*U*_iso_*/*U*_eq_	
Br1	0.19909 (4)	0.54881 (2)	0.167549 (17)	0.05758 (11)	
Br2	0.46179 (4)	0.08329 (2)	0.081708 (18)	0.06287 (11)	
Br3	−0.27413 (4)	1.16393 (2)	0.23629 (2)	0.06781 (12)	
O1	0.3515 (2)	0.55570 (16)	−0.23976 (11)	0.0581 (5)	
O2	0.7806 (2)	−0.26508 (16)	0.47575 (11)	0.0566 (5)	
O3	0.1415 (2)	0.68795 (15)	0.26399 (12)	0.0577 (5)	
N1	0.2480 (2)	0.50577 (15)	−0.06225 (12)	0.0356 (5)	
N2	−0.0973 (4)	0.7573 (2)	0.02687 (16)	0.0707 (8)	
N3	0.6118 (2)	−0.15356 (15)	0.30490 (12)	0.0353 (5)	
N4	0.2182 (4)	0.1597 (2)	0.23731 (19)	0.0856 (10)	
N5	0.0605 (3)	0.90135 (17)	0.24767 (13)	0.0424 (5)	
N6	−0.2081 (4)	1.0176 (3)	0.46003 (18)	0.0832 (9)	
C1	0.1094 (4)	0.7134 (3)	−0.36610 (17)	0.0610 (8)	
H1A	0.0176	0.7695	−0.3929	0.073*	
H1B	0.2148	0.7308	−0.3658	0.073*	
C2	0.1090 (4)	0.6175 (3)	−0.37514 (18)	0.0628 (8)	
H2A	0.2142	0.5755	−0.3803	0.075*	
H2B	0.0169	0.6142	−0.4074	0.075*	
C3	0.0760 (3)	0.6309 (2)	−0.29139 (16)	0.0497 (7)	
H3A	−0.0378	0.6372	−0.2744	0.060*	
C4	0.2085 (3)	0.5798 (2)	−0.22413 (16)	0.0434 (6)	
C5	0.1577 (3)	0.5688 (2)	−0.13826 (15)	0.0384 (6)	
C6	0.0458 (3)	0.6420 (2)	−0.12020 (16)	0.0439 (6)	
H6A	−0.0318	0.6928	−0.1586	0.053*	
C7	0.0660 (3)	0.6288 (2)	−0.03475 (15)	0.0411 (6)	
C8	−0.0227 (4)	0.6977 (2)	0.00283 (17)	0.0491 (7)	
C9	0.1922 (3)	0.54384 (18)	0.00154 (15)	0.0362 (6)	
C10	0.2650 (3)	0.4931 (2)	0.08306 (16)	0.0438 (6)	
C11	0.3832 (4)	0.4075 (2)	0.10048 (18)	0.0573 (8)	
H11A	0.4347	0.3754	0.1541	0.069*	
C12	0.4265 (4)	0.3683 (2)	0.03594 (19)	0.0568 (8)	
H12A	0.5051	0.3081	0.0485	0.068*	
C13	0.3596 (3)	0.4136 (2)	−0.04408 (17)	0.0438 (6)	
C14	0.3917 (4)	0.3656 (2)	−0.10947 (19)	0.0585 (8)	
H14A	0.4715	0.3030	−0.0872	0.088*	
H14B	0.4334	0.4089	−0.1574	0.088*	
H14C	0.2910	0.3540	−0.1261	0.088*	
C15	0.5985 (4)	−0.3051 (3)	0.62270 (17)	0.0666 (9)	
H15A	0.5205	−0.3340	0.6607	0.080*	
H15B	0.7051	−0.3503	0.6255	0.080*	
C16	0.5944 (5)	−0.1992 (3)	0.6058 (2)	0.0767 (11)	
H16A	0.6984	−0.1792	0.5984	0.092*	
H16B	0.5139	−0.1629	0.6335	0.092*	
C17	0.5321 (3)	−0.2299 (2)	0.53902 (16)	0.0514 (7)	
H17A	0.4123	−0.2129	0.5276	0.062*	
C18	0.6347 (3)	−0.2247 (2)	0.46623 (15)	0.0420 (6)	
C19	0.5511 (3)	−0.15996 (19)	0.38451 (15)	0.0383 (6)	
C20	0.4309 (3)	−0.0747 (2)	0.37356 (16)	0.0436 (6)	
H20A	0.3686	−0.0599	0.4159	0.052*	
C21	0.4158 (3)	−0.01316 (19)	0.28948 (15)	0.0412 (6)	
C22	0.3082 (4)	0.0835 (2)	0.25739 (18)	0.0535 (7)	
C23	0.5298 (3)	−0.06160 (19)	0.24619 (15)	0.0361 (6)	
C24	0.5740 (3)	−0.0387 (2)	0.16214 (15)	0.0423 (6)	
C25	0.6904 (3)	−0.1037 (2)	0.13926 (16)	0.0472 (7)	
H25A	0.7250	−0.0863	0.0847	0.057*	
C26	0.7591 (3)	−0.1979 (2)	0.19828 (16)	0.0457 (7)	
H26A	0.8365	−0.2433	0.1813	0.055*	
C27	0.7170 (3)	−0.22544 (19)	0.27943 (16)	0.0403 (6)	
C28	0.7673 (4)	−0.3313 (2)	0.33769 (19)	0.0589 (8)	
H28A	0.8405	−0.3708	0.3105	0.088*	
H28B	0.6712	−0.3577	0.3523	0.088*	
H28C	0.8226	−0.3334	0.3877	0.088*	
C29	0.3862 (4)	0.5520 (3)	0.3895 (2)	0.0724 (10)	
H29A	0.3983	0.5487	0.3332	0.087*	
H29B	0.4868	0.5249	0.4256	0.087*	
C30	0.2311 (4)	0.5385 (3)	0.4284 (2)	0.0750 (10)	
H30A	0.2362	0.5032	0.4885	0.090*	
H30B	0.1476	0.5270	0.3962	0.090*	
C31	0.2666 (4)	0.6409 (2)	0.39932 (19)	0.0583 (8)	
H31A	0.2951	0.6665	0.4425	0.070*	
C32	0.1748 (3)	0.7145 (2)	0.32149 (17)	0.0474 (7)	
C33	0.1166 (3)	0.8167 (2)	0.31987 (16)	0.0437 (6)	
C34	0.0518 (3)	0.8423 (2)	0.38694 (17)	0.0473 (7)	
H34A	0.0720	0.8011	0.4429	0.057*	
C35	−0.0480 (3)	0.9381 (2)	0.35932 (17)	0.0463 (7)	
C36	−0.1394 (4)	0.9854 (2)	0.41319 (18)	0.0569 (8)	
C37	−0.0449 (3)	0.9762 (2)	0.27152 (16)	0.0434 (6)	
C38	−0.1185 (3)	1.0661 (2)	0.20829 (17)	0.0493 (7)	
C39	−0.0805 (4)	1.0834 (2)	0.12739 (19)	0.0594 (8)	
H39A	−0.1322	1.1424	0.0854	0.071*	
C40	0.0386 (4)	1.0104 (3)	0.10818 (18)	0.0615 (9)	
H40A	0.0698	1.0244	0.0531	0.074*	
C41	0.1104 (4)	0.9210 (2)	0.16544 (17)	0.0510 (7)	
C42	0.2461 (4)	0.8478 (3)	0.1459 (2)	0.0676 (9)	
H42A	0.2645	0.8719	0.0870	0.101*	
H42B	0.2167	0.7845	0.1598	0.101*	
H42C	0.3450	0.8396	0.1781	0.101*	

### Atomic displacement parameters (Å^2^)

**Table:**

	*U*^11^	*U*^22^	*U*^33^	*U*^12^	*U*^13^	*U*^23^
Br1	0.0736 (2)	0.0676 (2)	0.03688 (16)	−0.02012 (16)	0.00100 (13)	−0.02217 (15)
Br2	0.0887 (2)	0.0501 (2)	0.03824 (17)	−0.01080 (16)	−0.01259 (14)	−0.00278 (14)
Br3	0.0797 (2)	0.0513 (2)	0.0705 (2)	−0.01124 (16)	0.00471 (17)	−0.02090 (17)
O1	0.0445 (11)	0.0798 (15)	0.0409 (11)	−0.0090 (10)	0.0077 (8)	−0.0129 (10)
O2	0.0390 (11)	0.0724 (14)	0.0430 (11)	−0.0031 (10)	0.0027 (8)	−0.0065 (10)
O3	0.0696 (13)	0.0582 (13)	0.0530 (12)	−0.0119 (10)	−0.0043 (10)	−0.0303 (11)
N1	0.0396 (11)	0.0337 (12)	0.0327 (11)	−0.0100 (9)	0.0020 (8)	−0.0095 (9)
N2	0.092 (2)	0.0556 (17)	0.0534 (16)	−0.0006 (15)	0.0230 (14)	−0.0161 (14)
N3	0.0376 (11)	0.0353 (12)	0.0320 (11)	−0.0089 (9)	0.0011 (8)	−0.0097 (9)
N4	0.094 (2)	0.064 (2)	0.079 (2)	0.0265 (18)	−0.0247 (17)	−0.0258 (17)
N5	0.0517 (13)	0.0485 (14)	0.0366 (12)	−0.0235 (11)	0.0095 (10)	−0.0194 (11)
N6	0.090 (2)	0.097 (2)	0.0611 (18)	0.0017 (18)	0.0166 (16)	−0.0417 (18)
C1	0.0641 (19)	0.066 (2)	0.0413 (16)	−0.0130 (16)	−0.0015 (14)	−0.0040 (15)
C2	0.071 (2)	0.077 (2)	0.0420 (16)	−0.0137 (17)	−0.0046 (14)	−0.0242 (16)
C3	0.0417 (15)	0.069 (2)	0.0341 (14)	−0.0135 (14)	0.0028 (11)	−0.0119 (14)
C4	0.0454 (16)	0.0485 (17)	0.0349 (14)	−0.0147 (13)	0.0044 (11)	−0.0102 (12)
C5	0.0404 (14)	0.0432 (15)	0.0310 (13)	−0.0121 (12)	0.0019 (10)	−0.0101 (12)
C6	0.0428 (14)	0.0448 (16)	0.0357 (14)	−0.0060 (12)	0.0020 (11)	−0.0056 (12)
C7	0.0440 (14)	0.0415 (16)	0.0372 (14)	−0.0119 (12)	0.0103 (11)	−0.0117 (12)
C8	0.0580 (17)	0.0463 (18)	0.0387 (15)	−0.0134 (14)	0.0130 (13)	−0.0086 (14)
C9	0.0438 (14)	0.0336 (14)	0.0330 (13)	−0.0150 (11)	0.0060 (11)	−0.0099 (11)
C10	0.0559 (16)	0.0429 (16)	0.0349 (14)	−0.0179 (13)	−0.0001 (12)	−0.0118 (12)
C11	0.070 (2)	0.0491 (19)	0.0443 (16)	−0.0070 (16)	−0.0140 (14)	−0.0085 (14)
C12	0.0640 (19)	0.0373 (17)	0.0594 (19)	−0.0004 (14)	−0.0101 (15)	−0.0109 (15)
C13	0.0476 (15)	0.0355 (15)	0.0471 (15)	−0.0081 (12)	0.0037 (12)	−0.0136 (13)
C14	0.073 (2)	0.0442 (18)	0.0598 (19)	−0.0086 (15)	0.0099 (15)	−0.0230 (15)
C15	0.064 (2)	0.079 (3)	0.0362 (16)	−0.0077 (17)	0.0092 (14)	0.0007 (16)
C16	0.075 (2)	0.109 (3)	0.057 (2)	−0.019 (2)	0.0127 (17)	−0.044 (2)
C17	0.0418 (15)	0.066 (2)	0.0373 (15)	−0.0086 (13)	0.0050 (11)	−0.0093 (14)
C18	0.0421 (16)	0.0455 (16)	0.0354 (14)	−0.0127 (13)	0.0024 (11)	−0.0084 (12)
C19	0.0380 (13)	0.0415 (15)	0.0339 (13)	−0.0099 (11)	0.0035 (10)	−0.0105 (11)
C20	0.0413 (14)	0.0484 (17)	0.0392 (14)	−0.0051 (12)	0.0008 (11)	−0.0161 (13)
C21	0.0415 (14)	0.0402 (16)	0.0396 (14)	−0.0041 (12)	−0.0051 (11)	−0.0138 (12)
C22	0.0583 (18)	0.0524 (19)	0.0452 (16)	0.0007 (15)	−0.0118 (13)	−0.0189 (14)
C23	0.0410 (14)	0.0345 (14)	0.0343 (13)	−0.0121 (11)	−0.0044 (10)	−0.0109 (11)
C24	0.0536 (16)	0.0417 (16)	0.0317 (13)	−0.0163 (13)	−0.0067 (11)	−0.0088 (12)
C25	0.0541 (16)	0.0606 (19)	0.0333 (14)	−0.0217 (14)	0.0055 (12)	−0.0189 (14)
C26	0.0467 (15)	0.0482 (17)	0.0463 (16)	−0.0106 (13)	0.0052 (12)	−0.0219 (14)
C27	0.0415 (14)	0.0380 (15)	0.0428 (14)	−0.0098 (12)	0.0037 (11)	−0.0153 (12)
C28	0.072 (2)	0.0379 (17)	0.0585 (18)	−0.0051 (15)	0.0139 (15)	−0.0119 (14)
C29	0.067 (2)	0.075 (2)	0.064 (2)	0.0030 (18)	−0.0076 (17)	−0.0227 (19)
C30	0.080 (2)	0.061 (2)	0.071 (2)	−0.0128 (18)	0.0024 (18)	−0.0066 (18)
C31	0.0689 (19)	0.057 (2)	0.0499 (17)	−0.0105 (16)	−0.0032 (14)	−0.0215 (15)
C32	0.0485 (16)	0.0537 (18)	0.0457 (16)	−0.0161 (13)	0.0050 (12)	−0.0217 (14)
C33	0.0505 (15)	0.0497 (17)	0.0372 (14)	−0.0220 (13)	0.0075 (12)	−0.0166 (13)
C34	0.0550 (16)	0.0552 (19)	0.0366 (14)	−0.0209 (14)	0.0038 (12)	−0.0168 (13)
C35	0.0503 (16)	0.0552 (18)	0.0422 (15)	−0.0200 (14)	0.0099 (12)	−0.0235 (14)
C36	0.0617 (19)	0.063 (2)	0.0474 (17)	−0.0136 (16)	0.0080 (14)	−0.0223 (16)
C37	0.0486 (15)	0.0489 (17)	0.0413 (15)	−0.0217 (13)	0.0074 (12)	−0.0201 (13)
C38	0.0589 (17)	0.0450 (17)	0.0500 (17)	−0.0239 (14)	0.0070 (13)	−0.0164 (14)
C39	0.076 (2)	0.054 (2)	0.0504 (18)	−0.0285 (17)	0.0078 (15)	−0.0124 (15)
C40	0.081 (2)	0.078 (2)	0.0362 (16)	−0.0414 (19)	0.0160 (15)	−0.0181 (17)
C41	0.0628 (18)	0.064 (2)	0.0403 (15)	−0.0345 (16)	0.0160 (13)	−0.0244 (16)
C42	0.079 (2)	0.082 (3)	0.060 (2)	−0.0350 (19)	0.0335 (17)	−0.0380 (19)

### Geometric parameters (Å, º)

**Table:**

Br1—C10	1.883 (3)	C16—C17	1.489 (4)
Br2—C24	1.887 (3)	C16—H16A	0.9700
Br3—C38	1.879 (3)	C16—H16B	0.9700
O1—C4	1.215 (3)	C17—C18	1.476 (4)
O2—C18	1.213 (3)	C17—H17A	0.9800
O3—C32	1.219 (3)	C18—C19	1.467 (3)
N1—C13	1.389 (3)	C19—C20	1.368 (4)
N1—C9	1.398 (3)	C20—C21	1.394 (4)
N1—C5	1.413 (3)	C20—H20A	0.9300
N2—C8	1.135 (3)	C21—C23	1.397 (3)
N3—C27	1.387 (3)	C21—C22	1.420 (4)
N3—C23	1.403 (3)	C23—C24	1.409 (3)
N3—C19	1.410 (3)	C24—C25	1.343 (4)
N4—C22	1.136 (4)	C25—C26	1.398 (4)
N5—C41	1.398 (3)	C25—H25A	0.9300
N5—C37	1.405 (3)	C26—C27	1.358 (4)
N5—C33	1.408 (3)	C26—H26A	0.9300
N6—C36	1.134 (4)	C27—C28	1.494 (4)
C1—C2	1.458 (5)	C28—H28A	0.9600
C1—C3	1.497 (4)	C28—H28B	0.9600
C1—H1A	0.9700	C28—H28C	0.9600
C1—H1B	0.9700	C29—C30	1.464 (5)
C2—C3	1.505 (4)	C29—C31	1.501 (4)
C2—H2A	0.9700	C29—H29A	0.9700
C2—H2B	0.9700	C29—H29B	0.9700
C3—C4	1.476 (4)	C30—C31	1.504 (5)
C3—H3A	0.9800	C30—H30A	0.9700
C4—C5	1.471 (3)	C30—H30B	0.9700
C5—C6	1.363 (4)	C31—C32	1.480 (4)
C6—C7	1.401 (3)	C31—H31A	0.9800
C6—H6A	0.9300	C32—C33	1.450 (4)
C7—C9	1.394 (3)	C33—C34	1.373 (4)
C7—C8	1.425 (4)	C34—C35	1.382 (4)
C9—C10	1.401 (3)	C34—H34A	0.9300
C10—C11	1.350 (4)	C35—C37	1.402 (4)
C11—C12	1.399 (4)	C35—C36	1.429 (4)
C11—H11A	0.9300	C37—C38	1.400 (4)
C12—C13	1.358 (4)	C38—C39	1.351 (4)
C12—H12A	0.9300	C39—C40	1.401 (5)
C13—C14	1.486 (4)	C39—H39A	0.9300
C14—H14A	0.9600	C40—C41	1.349 (4)
C14—H14B	0.9600	C40—H40A	0.9300
C14—H14C	0.9600	C41—C42	1.484 (4)
C15—C16	1.466 (5)	C42—H42A	0.9600
C15—C17	1.492 (4)	C42—H42B	0.9600
C15—H15A	0.9700	C42—H42C	0.9600
C15—H15B	0.9700		
			
C13—N1—C9	121.0 (2)	C20—C19—C18	123.7 (2)
C13—N1—C5	130.0 (2)	N3—C19—C18	126.3 (2)
C9—N1—C5	108.59 (19)	C19—C20—C21	109.8 (2)
C27—N3—C23	121.0 (2)	C19—C20—H20A	125.1
C27—N3—C19	130.0 (2)	C21—C20—H20A	125.1
C23—N3—C19	108.50 (19)	C20—C21—C23	107.7 (2)
C41—N5—C37	121.0 (2)	C20—C21—C22	124.3 (2)
C41—N5—C33	129.6 (2)	C23—C21—C22	127.8 (2)
C37—N5—C33	108.9 (2)	N4—C22—C21	175.1 (3)
C2—C1—C3	61.2 (2)	C21—C23—N3	107.0 (2)
C2—C1—H1A	117.6	C21—C23—C24	134.9 (2)
C3—C1—H1A	117.6	N3—C23—C24	118.1 (2)
C2—C1—H1B	117.6	C25—C24—C23	120.6 (2)
C3—C1—H1B	117.6	C25—C24—Br2	120.6 (2)
H1A—C1—H1B	114.8	C23—C24—Br2	118.7 (2)
C1—C2—C3	60.7 (2)	C24—C25—C26	119.4 (2)
C1—C2—H2A	117.7	C24—C25—H25A	120.3
C3—C2—H2A	117.7	C26—C25—H25A	120.3
C1—C2—H2B	117.7	C27—C26—C25	122.5 (2)
C3—C2—H2B	117.7	C27—C26—H26A	118.7
H2A—C2—H2B	114.8	C25—C26—H26A	118.7
C4—C3—C1	117.4 (2)	C26—C27—N3	117.6 (2)
C4—C3—C2	116.9 (3)	C26—C27—C28	121.9 (2)
C1—C3—C2	58.1 (2)	N3—C27—C28	120.2 (2)
C4—C3—H3A	117.1	C27—C28—H28A	109.5
C1—C3—H3A	117.1	C27—C28—H28B	109.5
C2—C3—H3A	117.1	H28A—C28—H28B	109.5
O1—C4—C5	122.6 (2)	C27—C28—H28C	109.5
O1—C4—C3	121.7 (2)	H28A—C28—H28C	109.5
C5—C4—C3	115.5 (2)	H28B—C28—H28C	109.5
C6—C5—N1	107.1 (2)	C30—C29—C31	60.9 (2)
C6—C5—C4	122.3 (2)	C30—C29—H29A	117.7
N1—C5—C4	127.0 (2)	C31—C29—H29A	117.7
C5—C6—C7	109.3 (2)	C30—C29—H29B	117.7
C5—C6—H6A	125.3	C31—C29—H29B	117.7
C7—C6—H6A	125.3	H29A—C29—H29B	114.8
C9—C7—C6	108.0 (2)	C29—C30—C31	60.8 (2)
C9—C7—C8	128.5 (2)	C29—C30—H30A	117.7
C6—C7—C8	123.4 (2)	C31—C30—H30A	117.7
N2—C8—C7	174.9 (3)	C29—C30—H30B	117.7
C7—C9—N1	107.0 (2)	C31—C30—H30B	117.7
C7—C9—C10	134.4 (2)	H30A—C30—H30B	114.8
N1—C9—C10	118.6 (2)	C32—C31—C29	116.1 (3)
C11—C10—C9	120.9 (2)	C32—C31—C30	116.7 (3)
C11—C10—Br1	120.5 (2)	C29—C31—C30	58.3 (2)
C9—C10—Br1	118.6 (2)	C32—C31—H31A	117.5
C10—C11—C12	118.4 (3)	C29—C31—H31A	117.5
C10—C11—H11A	120.8	C30—C31—H31A	117.5
C12—C11—H11A	120.8	O3—C32—C33	122.1 (3)
C13—C12—C11	123.4 (3)	O3—C32—C31	120.6 (3)
C13—C12—H12A	118.3	C33—C32—C31	117.0 (2)
C11—C12—H12A	118.3	C34—C33—N5	106.4 (2)
C12—C13—N1	117.3 (2)	C34—C33—C32	124.1 (3)
C12—C13—C14	122.8 (3)	N5—C33—C32	125.8 (2)
N1—C13—C14	119.8 (2)	C33—C34—C35	110.3 (2)
C13—C14—H14A	109.5	C33—C34—H34A	124.9
C13—C14—H14B	109.5	C35—C34—H34A	124.9
H14A—C14—H14B	109.5	C34—C35—C37	107.9 (2)
C13—C14—H14C	109.5	C34—C35—C36	124.2 (3)
H14A—C14—H14C	109.5	C37—C35—C36	127.9 (3)
H14B—C14—H14C	109.5	N6—C36—C35	175.7 (4)
C16—C15—C17	60.4 (2)	C38—C37—C35	135.4 (3)
C16—C15—H15A	117.7	C38—C37—N5	118.1 (2)
C17—C15—H15A	117.7	C35—C37—N5	106.6 (2)
C16—C15—H15B	117.7	C39—C38—C37	121.1 (3)
C17—C15—H15B	117.7	C39—C38—Br3	119.4 (2)
H15A—C15—H15B	114.9	C37—C38—Br3	119.4 (2)
C15—C16—C17	60.7 (2)	C38—C39—C40	118.5 (3)
C15—C16—H16A	117.7	C38—C39—H39A	120.8
C17—C16—H16A	117.7	C40—C39—H39A	120.8
C15—C16—H16B	117.7	C41—C40—C39	123.4 (3)
C17—C16—H16B	117.7	C41—C40—H40A	118.3
H16A—C16—H16B	114.8	C39—C40—H40A	118.3
C18—C17—C16	116.1 (3)	C40—C41—N5	117.2 (3)
C18—C17—C15	118.6 (2)	C40—C41—C42	122.8 (3)
C16—C17—C15	58.9 (2)	N5—C41—C42	119.8 (3)
C18—C17—H17A	116.9	C41—C42—H42A	109.5
C16—C17—H17A	116.9	C41—C42—H42B	109.5
C15—C17—H17A	116.9	H42A—C42—H42B	109.5
O2—C18—C19	123.6 (2)	C41—C42—H42C	109.5
O2—C18—C17	121.1 (2)	H42A—C42—H42C	109.5
C19—C18—C17	115.0 (2)	H42B—C42—H42C	109.5
C20—C19—N3	106.9 (2)		
			
C2—C1—C3—C4	106.1 (3)	C20—C21—C23—C24	−179.8 (3)
C1—C2—C3—C4	−107.0 (3)	C22—C21—C23—C24	−3.0 (5)
C1—C3—C4—O1	−43.4 (4)	C27—N3—C23—C21	171.1 (2)
C2—C3—C4—O1	22.7 (4)	C19—N3—C23—C21	−1.6 (3)
C1—C3—C4—C5	131.3 (3)	C27—N3—C23—C24	−8.4 (3)
C2—C3—C4—C5	−162.6 (3)	C19—N3—C23—C24	178.9 (2)
C13—N1—C5—C6	170.1 (2)	C21—C23—C24—C25	−178.8 (3)
C9—N1—C5—C6	−1.7 (3)	N3—C23—C24—C25	0.5 (4)
C13—N1—C5—C4	−31.1 (4)	C21—C23—C24—Br2	−0.7 (4)
C9—N1—C5—C4	157.1 (3)	N3—C23—C24—Br2	178.63 (16)
O1—C4—C5—C6	135.9 (3)	C23—C24—C25—C26	4.8 (4)
C3—C4—C5—C6	−38.7 (4)	Br2—C24—C25—C26	−173.3 (2)
O1—C4—C5—N1	−20.0 (4)	C24—C25—C26—C27	−2.4 (4)
C3—C4—C5—N1	165.4 (3)	C25—C26—C27—N3	−5.3 (4)
N1—C5—C6—C7	1.7 (3)	C25—C26—C27—C28	168.8 (3)
C4—C5—C6—C7	−158.3 (2)	C23—N3—C27—C26	10.7 (3)
C5—C6—C7—C9	−1.1 (3)	C19—N3—C27—C26	−178.4 (2)
C5—C6—C7—C8	174.6 (2)	C23—N3—C27—C28	−163.5 (2)
C6—C7—C9—N1	0.0 (3)	C19—N3—C27—C28	7.5 (4)
C8—C7—C9—N1	−175.4 (3)	C30—C29—C31—C32	−106.6 (3)
C6—C7—C9—C10	−178.9 (3)	C29—C30—C31—C32	105.6 (3)
C8—C7—C9—C10	5.8 (5)	C29—C31—C32—O3	30.5 (4)
C13—N1—C9—C7	−171.7 (2)	C30—C31—C32—O3	−35.4 (4)
C5—N1—C9—C7	1.1 (3)	C29—C31—C32—C33	−154.0 (3)
C13—N1—C9—C10	7.4 (3)	C30—C31—C32—C33	140.1 (3)
C5—N1—C9—C10	−179.9 (2)	C41—N5—C33—C34	168.7 (3)
C7—C9—C10—C11	176.7 (3)	C37—N5—C33—C34	−2.8 (3)
N1—C9—C10—C11	−2.0 (4)	C41—N5—C33—C32	−32.5 (4)
C7—C9—C10—Br1	−5.0 (4)	C37—N5—C33—C32	156.0 (2)
N1—C9—C10—Br1	176.22 (17)	O3—C32—C33—C34	135.6 (3)
C9—C10—C11—C12	−2.6 (4)	C31—C32—C33—C34	−39.8 (4)
Br1—C10—C11—C12	179.1 (2)	O3—C32—C33—N5	−19.6 (4)
C10—C11—C12—C13	2.2 (5)	C31—C32—C33—N5	165.0 (3)
C11—C12—C13—N1	2.9 (4)	N5—C33—C34—C35	2.4 (3)
C11—C12—C13—C14	−172.6 (3)	C32—C33—C34—C35	−156.9 (3)
C9—N1—C13—C12	−7.7 (4)	C33—C34—C35—C37	−1.1 (3)
C5—N1—C13—C12	−178.7 (3)	C33—C34—C35—C36	177.2 (3)
C9—N1—C13—C14	167.9 (2)	C34—C35—C37—C38	179.6 (3)
C5—N1—C13—C14	−3.1 (4)	C36—C35—C37—C38	1.4 (5)
C15—C16—C17—C18	−109.1 (3)	C34—C35—C37—N5	−0.7 (3)
C16—C15—C17—C18	104.8 (3)	C36—C35—C37—N5	−178.9 (3)
C16—C17—C18—O2	52.5 (4)	C41—N5—C37—C38	9.5 (4)
C15—C17—C18—O2	−14.7 (4)	C33—N5—C37—C38	−178.1 (2)
C16—C17—C18—C19	−121.7 (3)	C41—N5—C37—C35	−170.2 (2)
C15—C17—C18—C19	171.1 (3)	C33—N5—C37—C35	2.2 (3)
C27—N3—C19—C20	−170.0 (2)	C35—C37—C38—C39	175.0 (3)
C23—N3—C19—C20	1.8 (3)	N5—C37—C38—C39	−4.7 (4)
C27—N3—C19—C18	29.4 (4)	C35—C37—C38—Br3	−5.5 (4)
C23—N3—C19—C18	−158.8 (2)	N5—C37—C38—Br3	174.84 (18)
O2—C18—C19—C20	−141.3 (3)	C37—C38—C39—C40	−1.9 (4)
C17—C18—C19—C20	32.7 (4)	Br3—C38—C39—C40	178.6 (2)
O2—C18—C19—N3	16.2 (4)	C38—C39—C40—C41	4.2 (5)
C17—C18—C19—N3	−169.8 (3)	C39—C40—C41—N5	0.5 (5)
N3—C19—C20—C21	−1.2 (3)	C39—C40—C41—C42	−174.8 (3)
C18—C19—C20—C21	160.0 (2)	C37—N5—C41—C40	−7.4 (4)
C19—C20—C21—C23	0.2 (3)	C33—N5—C41—C40	−178.1 (3)
C19—C20—C21—C22	−176.7 (3)	C37—N5—C41—C42	168.1 (3)
C20—C21—C23—N3	0.9 (3)	C33—N5—C41—C42	−2.6 (4)
C22—C21—C23—N3	177.6 (3)		

### Hydrogen-bond geometry (Å, º)

**Table:**

*D*—H···*A*	*D*—H	H···*A*	*D*···*A*	*D*—H···*A*
C14—H14*B*···O1	0.96	2.09	2.857 (4)	136
C28—H28*C*···O2	0.96	2.04	2.823 (4)	138
C42—H42*B*···O3	0.96	2.04	2.814 (4)	136

## References

[bb1] Allen, F. H., Kennard, O., Watson, D. G., Brammer, L., Orpen, A. G. & Taylor, R. (1987). *J. Chem. Soc. Perkin Trans. 2*, pp. S1–19.

[bb2] Bruker (2006). *APEX2* and *SAINT* Bruker AXS Inc., Madison, Wisconsin, USA.

[bb3] Sheldrick, G. M. (1997). *SADABS* University of Göttingen, Germany.

[bb4] Sheldrick, G. M. (2008). *Acta Cryst.* A**64**, 112–122. 10.1107/S0108767307043930 18156677

[bb5] Shen, Y. M., Grampp, G., Leesakul, N., Hu, H. W. & Xu, J. H. (2007). *Eur. J. Org. Chem.* pp. 3718–3726.

[bb6] Shen, Y. M., Lv, P. C., Chen, W., Liu, P. G., Zhang, M. Z. & Zhu, H. L. (2010). *Eur. J. Med. Chem.* **45**, 3184–3190. 10.1016/j.ejmech.2010.02.056 20304535

[bb7] Sippl, W. (2002). *Bioorg. Med. Chem.* **10**, 3741–3755. 10.1016/s0968-0896(02)00375-9 12413831

[bb8] Sriram, D., Yogeeswari, P., Thirumurugan, R. & Bal, T. R. (2005). *Nat. Prod. Res.* **19**, 393–412. 10.1080/14786410412331299005 15938148

[bb9] Wu, X. W., Zu-Ping Wu, Z. P., Wang, L. X., Zhang, H. B., Chen, J. W., Zhang, W., Gu, L. Q., Huang, Z. S. & An, L. K. (2011). *Eur. J. Med. Chem.* **46**, 4625–4633. 10.1016/j.ejmech.2011.07.042 21839550

